# Supplementary material for: Controlled emission time statistics of a dynamic single-electron transistor
Source: Sci Adv. 2021 Jan 6;7(2):eabe0793. doi: 10.1126/sciadv.abe0793 (PMC7787478; doi:10.1126/sciadv.abe0793)
Supplement: http://advances.sciencemag.org/cgi/content/full/7/2/eabe0793/DC1 [file supp_7_2_eabe0793__index.html]

Science Advances | Science AdvancesAAASSearchScience AdvancesMenu

## Supplementary Materials

# Controlled emission time statistics of a dynamic single-electron transistor

Fredrik Brange, Adrian Schmidt, Johannes C. Bayer, Timo Wagner, Christian Flindt, Rolf J. Haug

Download Supplement

**This PDF file includes:**

- Calculations of waiting time distributions

**Files in this Data Supplement:**

- Adobe PDF - abe0793\_SM.pdf
