# Supplementary material for: Controlled emission time statistics of a dynamic single-electron transistor
Source: Sci Adv. 2021 Jan 6;7(2):eabe0793. doi: 10.1126/sciadv.abe0793 (PMC7787478; doi:10.1126/sciadv.abe0793)
Supplement: http://advances.sciencemag.org/cgi/content/full/7/2/eabe0793/DC1 [file supp_7_2_eabe0793__1.pdf]

Science Advances
AAAS

advances.sciencemag.org/cgi/content/full/7/2/eabe0793/DC1

Supplementary Materials for

Controlled emission time statistics of a dynamic single-electron transistor

Fredrik Brange, Adrian Schmidt, Johannes C. Bayer, Timo Wagner, Christian Flindt*, Rolf J. Haug*

*Corresponding author. Email: haug@nano.uni-hannover.de (R.J.H.); christian.flindt@aalto.fi (C.F.)

Published 6 January 2021, *Sci. Adv.* **7**, eabe0793 (2021)
DOI: 10.1126/sciadv.abe0793

This PDF file includes:

Calculations of waiting time distributions

Supplementary material:

CALCULATIONS OF WAITING TIME DISTRIBUTIONS

Numerical calculations

To calculate the distribution of waiting times, we follow the Materials and Methods section and Ref. 16 of the main text. We first compute the idle-time probability as $\Pi(\tau, t_0) = \langle 1|\mathbf{U}_0(\tau{+}t_0, t_0)\,|p_s(t_0)\rangle$ with $\mathbf{U}_0(t, t_0) = \hat{T}\{e^{\int_{t_0}^{t} dt_1 \mathbf{L}_0(t_1)}\}$, where $\hat{T}$ is the time-ordering operator and $\langle 1| \equiv (1, 1)$. For the periodic state, $|p_s(t_0)\rangle = |p_s(t_0 + T)\rangle$, we find

$$p_1(t_0) = \frac{e^{-\int_{t_0}^{t_0+T} dt_1(\Gamma_i(t_1)+\Gamma_o(t_1))}}{1 - e^{-\int_{t_0}^{t_0+T} dt_1(\Gamma_i(t_1)+\Gamma_o(t_1))}} \int_{t_0}^{t_0+T} dt_1 \Gamma_i(t_1) e^{\int_{t_0}^{t_1} dt_2[\Gamma_i(t_2)+\Gamma_o(t_2)]}. \tag{1}$$

with the normalization, $p_0(t_0) + p_1(t_0) = 1$, at all times. For the non-zero matrix elements of $\mathbf{U}_0(t, t_0)$, we have[16]

$$\begin{aligned} [\mathbf{U}_0(t, t_0)]_{11} &= \exp\left[-\int_{t_0}^{t} dt_1 \Gamma_i(t_1)\right], \qquad [\mathbf{U}_0(t, t_0)]_{22} = \exp\left[-\int_{t_0}^{t} dt_1 \Gamma_o(t_1)\right], \\ [\mathbf{U}_0(t, t_0)]_{21} &= \exp\left[-\int_{t_0}^{t} dt_1 \Gamma_o(t_1)\right] \int_{t_0}^{t} dt_1 \Gamma_i(t_1) \exp\left[\int_{t_0}^{t_1} dt_2 \left[\Gamma_o(t_2) - \Gamma_i(t_2)\right]\right], \end{aligned} \tag{2}$$

and thereby obtain

$$\Pi(\tau, t_0) = U_{11}(t_0 + \tau, t_0)[1 - p_1(t_0)] + U_{21}(t_0 + \tau, t_0)[1 - p_1(t_0)] + U_{22}(t_0 + \tau, t_0) p_1(t_0). \tag{3}$$

By evaluating these expressions numerically, we obtain the waiting time distribution as $\mathcal{W}(\tau) = \langle\tau\rangle \partial_\tau^2 \Pi(\tau)$ with $\langle\tau\rangle = -1/[\partial_\tau \Pi(\tau = 0)]$ and $\Pi(\tau) = \int_0^T dt_0 \Pi(\tau, t_0)/T$.

Zero-frequency limit

With constant rates, $\Gamma_i(t) = \Gamma_i$ and $\Gamma_o(t) = \Gamma_o$, Eqs. (1) and (2) are easily solved analytically, yielding

$$p_1^s(t_0, \Gamma_i, \Gamma_o) = \frac{\Gamma_i}{\Gamma_i + \Gamma_o}, \tag{4}$$

and

$$\begin{aligned} [\mathbf{U}_0(t, t_0, \Gamma_i)]_{11}^s &= \exp\left[-\Gamma_i(t - t_0)\right], \qquad [\mathbf{U}_0(t, t_0, \Gamma_o)]_{22}^s = \exp\left[-\Gamma_o(t - t_0)\right], \\ [\mathbf{U}_0(t, t_0, \Gamma_i, \Gamma_o)]_{21}^s &= \frac{\Gamma_i}{\Gamma_i - \Gamma_o}\left(e^{-\Gamma_o(t-t_0)} - e^{-\Gamma_i(t-t_0)}\right). \end{aligned} \tag{5}$$

We then find

$$\Pi_s(t, \Gamma_i, \Gamma_o) = \frac{e^{-\Gamma_o t}\Gamma_i^2 - e^{-\Gamma_i t}\Gamma_o^2}{\Gamma_i^2 - \Gamma_o^2}, \tag{6}$$

and

$$\mathcal{W}_s(\tau, \Gamma_i, \Gamma_o) = \frac{\Gamma_i \Gamma_o}{\Gamma_i - \Gamma_o}\left(e^{-\Gamma_o \tau} - e^{-\Gamma_i \tau}\right), \tag{7}$$

which is Eq. (1) of the main text.

High-frequency limit

For $\alpha_i, \alpha_o \ll 1$, we may treat the driving as a perturbation and expand all quantities to second order in the driving amplitudes (the first-order contribution to the waiting time distribution eventually vanishes). We start by expanding the tunneling rates as

$$\Gamma_i(t) = \Gamma_i \left[1 + \alpha_i \sin(2\pi f t) + \frac{1}{2}\alpha_i^2 \sin^2(2\pi f t)\right], \quad \Gamma_o(t) = \Gamma_o \left[1 - \alpha_o \sin(2\pi f t) + \frac{1}{2}\alpha_o^2 \sin^2(2\pi f t)\right]. \tag{8}$$

We note that the average values of the rates over a full driving period are

$$\overline{\Gamma}_i = \frac{1}{T}\int_0^T dt \Gamma_i(t) = \Gamma_i(1 + \alpha_i^2/4), \quad \overline{\Gamma}_o = \frac{1}{T}\int_0^T dt \Gamma_o(t) = \Gamma_o(1 + \alpha_o^2/4). \tag{9}$$

In principle, second-order perturbation theory allows us to derive an analytic expression for the waiting time distribution for all frequencies. However, given the lengthy expressions that the general derivation yields, we focus here on the high-frequency limit, $f \gg \Gamma_i, \Gamma_o$, where all expressions may be simplified considerably. Below we expand each quantity around its corresponding steady-state equivalent with the averaged rates $\overline{\Gamma}_i$ and $\overline{\Gamma}_o$ inserted. We neglect terms that are negligible in the high-frequency limit, $f \gg \Gamma_i, \Gamma_o$ and then find

$$p_1(t_0) = p_1^s(t_0, \overline{\Gamma}_i, \overline{\Gamma}_o)\left(1 - \frac{\Gamma_o}{2\pi f}\cos(2\pi f t_0)(\alpha_i + \alpha_o)\right), \tag{10}$$

as well as

$$[\mathbf{U}_0(t,t_0)]_{11} = [\mathbf{U}_0(t,t_0,\overline{\Gamma}_i)]_{11}^s\left(1 + \gamma_i + \gamma_i^2/2\right), \quad [\mathbf{U}_0(t,t_0)]_{22} = [\mathbf{U}_0(t,t_0,\overline{\Gamma}_o)]_{22}^s\left(1 + \gamma_o + \gamma_o^2/2\right), \tag{11}$$

and

$$\begin{aligned}[\mathbf{U}_0(t,t_0)]_{21} =& [\mathbf{U}_0(t,t_0,\overline{\Gamma}_i,\overline{\Gamma}_o)]_{21}^s \Bigg(1 + \frac{\cos[2\pi f t]}{2\pi f}\left[\frac{e^{\Gamma_o t + \Gamma_i t_0}\alpha_i(\Gamma_i - \Gamma_o)}{e^{\Gamma_o t + \Gamma_i t_0} - e^{\Gamma_i t + \Gamma_o t_0}} - \alpha_o\Gamma_o\right] + \frac{1}{2(2\pi f)^2}\Bigg[\alpha_i\alpha_o\Gamma_i\Gamma_o\cos(2\pi f(t - t_0)) \\ &- \frac{2\alpha_i(\Gamma_i - \Gamma_o)\left(e^{\Gamma_o t + \Gamma_i t_0}\cos(2\pi f t) - e^{\Gamma_i t + \Gamma_o t_0}\cos(2\pi f t_0)\right)(\alpha_o\Gamma_o\cos(2\pi f t) + \alpha_i\Gamma_i\cos(2\pi f t_0))}{e^{\Gamma_o t + \Gamma_i t_0} - e^{\Gamma_i t + \Gamma_o t_0}}\Bigg]\Bigg),\end{aligned} \tag{12}$$

with $\gamma_x \equiv \frac{\alpha_x \Gamma_x}{2\pi f}(\cos[2\pi f t] - \cos[2\pi f t_0])$, $x = i, o$. In the expressions above for the matrix elements of $\mathbf{U}_0(t,t_0)$, we have omitted terms that eventually vanish when averaged over a period of the drive. By averaging the idle-time probability in Eq. (3) over a period of the drive, we find

$$\Pi(t) = \Pi_s(t, \overline{\Gamma}_i, \overline{\Gamma}_o)\left(1 - \frac{1}{2}\left(\frac{1}{2\pi f}\right)^2 \frac{\Gamma_i^2\Gamma_o^2\left(e^{\Gamma_i t} - e^{\Gamma_o t}\right)}{e^{\Gamma_i t}\Gamma_i^2 - e^{\Gamma_o t}\Gamma_o^2}\cos(2\pi f t)\,\alpha_o^2\right), \tag{13}$$

and we then arrive at the high-frequency expression for the waiting time distribution in Eq. (3) of the main text,

$$\mathcal{W}(\tau) = \mathcal{W}_s(\tau, \overline{\Gamma}_i, \overline{\Gamma}_o)\left[1 + \frac{\alpha_o^2}{2}\cos(2\pi f \tau)\right]. \tag{14}$$
